# Dysbiosis of fecal microbiota and high frequency of *Citrobacter, Klebsiella* spp., and Actinomycetes in patients with irritable bowel syndrome and gastroenteritis 

**Published:** 2016

**Authors:** Leila Ganji, Masoud Alebouyeh, Mohammad Hassan Shirazi, Seyed Saeed Eshraghi, Abbas Mirshafiey, Naser Ebrahimi Daryani, Mohammad Reza Zali

**Affiliations:** 1 *Department of Pathobiology, School of Public Health, University of Medical Sciences, Tehran, Iran*; 2 *Foodborne and Waterborne Diseases Research Center, Research Institute for Gastroenterology and Liver Diseases, Shahid Beheshti University of Medical Science, Tehran, Iran.*; 3 *Gastroenterology and Liver Diseases Research Center, Research Institute for Gastroenterology and Liver Diseases, Shahid Beheshti University of Medical Science, Tehran, Iran.*; 4 *Department of Gastroenterology and Hepatology, School of Medicine, Tehran University of Medical Sciences, Tehran, Iran *

**Keywords:** Dysbiosis, Irritable bowel syndrome, Gastroenteritis

## Abstract

**Aim:**

This study was aimed to characterize putative differences of fecal microbiota between irritable bowel syndrome (IBS) and gastroenteritis patients and healthy controls.

**Background:**

New evidence proposed that gut microbiota has a deep effect on the balance between health and disease.

**Patients and methods:**

The presence of *Clostridium difficile*, *Campylobacter *spp., Enterobacteriacea and Staphylococci were detected in the samples using selective and specific culture media. Microscopic examination of the samples was done to detect Actinomycetes, yeasts, Bifidobacteria, *Fusobacterium *spp*.*, as well as white blood cells, red blood cells, mucus and epithelial cells.

**Results:**

Results of this study showed relatively higher frequency of *Citrobacter* spp., *Lactobacilli*, and *Actinomycetes i*n the IBS patients. Elevated levels of WBC, RBC secretion, and increased amounts of *Klebsiella*, *Escherichia coli* and *Citrobacter* spp. were characterized in the patients with gastroenteritis compared with the control group.

**Conclusion:**

Depletion of gram positive cocci and gram negative bacilli also suggested dysbiosis of intestinal microbiota in these patients.

## Introduction

 The gut microbiota is a complex ecosystem that has a crucial role in regulating gut motility, metabolism, immune function, and intestinal barrier homeostasis (1). Beside these beneficial effects, results of new studies has been established that alterations in microbiota balance (dysbiosis) have a role in certain intestinal disorders, such as enteric infections, colorectal cancer, inflammatory bowel disease, irritable bowel syndrome (IBS), and even obesity (2). 

IBS is a very common gastrointestinal disorder with a prevalence of 10-20% in the world (3). Clinical symptoms of IBS are different, so there are three Rome criteria for detection of IBS. According to the Rome criteria II, patients have been divided into four subtypes, i.e. constipation-predominant (IBS-C), diarrhea- predominant IBS (D-IBS), as well as mixed- type IBS (M-IBS) and unsubtyped IBS (4). There are some evidences regarding the involvement of bacteria in the occurrence of IBS, which are rise mainly from epidemiological and clinical studies (5). Association of acute bacterial gastroenteritis and IBS, known as post infection IBS, has been documented in several studies. This association could be due to an increase in mucosal colonic permeability, visceral hypersensitivity, inflammation, and disturbance of mucosal barrier through these bacteria (6). Indirect evidence about the role of bacteria in the occurrence of IBS raised from results that showed positive effects of antimicrobials or probiotics and prebiotics on treatments of the patients (7). Although these evidences support noted association, there is no congruence about responsible bacteria and manners of their pathogenesis in these patients. In this research study, our objective was to characterize putative differences of fecal microbiota between IBS and gastroenteritis patients and healthy controls by applying culture-based techniques and microscopic examinations. These differences could potentially have a causal relationship with the disease outcome. 

## Patients and Methods

We characterized a diversity of main bacterial enteropathogens and common members of fecal microbiota in fecal samples of patients with IBS, gastroenteritis and volunteer control subjects. The mean age of patients with IBS, gastroenteritis and control group was 38.7, 41.3 and 37.9 years, respectively. To examine this diversity, the presence of *Clostridium difficile*, *Campylobacter *spp., members of Enterobacteriacea, *Pseudomonas* spp., *Proteus* spp. and *Staphylococci* were detected in the samples, using selective and specific culture media as described below. Microscopic examination of the samples was done to detect specific morphologic microbial and human cells for Actinomycetes, spore forming bacteria, yeasts, Bacteroidetes, Lactobacilli, Bifidobacteria, *Fusobacterium *spp*.*, as well as white blood cells, red blood cells, mucus and epithelial cells. Also, overgrowth cocci and gram negative bacilli in the absence of normal flora was determined in each sample. Exclusion criteria for IBS patients were included intestinal disturbance (celiac disease and lactose intolerance), severe systematic disease, usage of antibiotic, and pregnancy. In the control group and patients with gastroenteritis, usage of antibiotic was the main exclusion criteria. Nearly, one gram of all samples were homogenized in phosphate buffered saline buffer, pH 8, and analyzed microscopically for studying the diversity of bacterial population after the gram staining. To analyze any difference in the frequency of culturable microorganisms and their overgrowth, a total of 100 µl volume of the suspension was cultured on different culture media, including MacConky agar (Merck, Germany) as a selective medium for members of Enterobacteriacea, Brucella agar supplemented with sheep blood (5%), Campylobacter supplement for detection of *Campylobacter* spp., and Clostridium selective agar and Mannitol salt agar for isolation of Clostridia and Staphylococci, respectively. The growth of aerobic and anaerobic bacteria was analyzed at 37^o^C after 24-48h incubation under aerobic and anaerobic conditions (Anoxomat: MART Microbiology B.V. 0% O_2_, 10% H2, 10% CO_2_ and 80% N2). The growth of *Campylobacter* was studied after incubation of the inoculated plates at 42^o^C under microaerophilic conditions. Colony counts of the grown colonies were determined in each case. Suspicious colonies were identified by routine microbiological and biochemical tests. The study was approved by the ethical committee of Shahid Beheshti University of Medical Sciences.

**Statistical analysis**

The frequency and percentage were used for categorizing qualitative variables. Statistical analysis was performed with SPSS software (version 23, Co Ltd, Tokyo, Japan). Differences between the groups were analyzed by the Pearson Chi- square and Fischer’s exact test. The results were considered to be significant if *p*-values were ≤0.05. 

## Results

We investigated 80 IBS patients full filling Rome II criteria, including IBS-D (n = 18), IBS-C (n = 29), or IBS-M (n = 33), 80 patients with gastroenteritis and 50 healthy controls. There was no statistically significant difference between the three groups regarding the age (*p=*0.7). However, while no difference was observed for the occurrence of gastroenteritis, IBS was found in a higher frequency among the female compared with male patients (*p* value <0.001). Bloating was the most common symptom of IBS patients (92.5%) (Table 1). 

**Table 1:** Frequency of symptoms in patients with IBS and gastroenteritis

Type of symptom	IBS* (%)	Gastroenteritis (%)
Diarrhea	27 (33.75)	40 (50)
Constipation	28 (35)	-
Abdominal pain	65 (81.2)	-
Bloating	74 (92.5)	-
Mixed type (diarrhea&constipation)	25(31.25)	-
dysentery	-	5 (6.2)
fever	-	13 (16.2)
Total	80 (100)	80 (100)

* Irritable bowel syndrome

In the culture method, results showed relatively higher frequency of some members of Enterobacteriacea (such as *Escherichia coli* and* Citrobacter *spp.) in fecal samples of the IBS patients, while the frequency of gram positive cocci were decreased (*p* value <0.005). Also, there was a significant difference in the frequency of *E.coli* and *Klebsiella *spp. between patients with IBS and gastroenteritis (*p*-value<0.05). The same association was confirmed by the microscopic examination, since these patients showed dominancy of Enterobacteracea in their smears. Indeed, these results showed a higher frequency of *Lactobacillus* and Actinomycetes among the IBS patients (*p* value <0.005). Although there was no significant difference in fecal bacteria between IBS patients were divided into three groups (Table 2). Compared with the control and Patients with IBS group, gastroenteritis patients had a significantly higher prevalence of WBC, RBC secretion and Gram-positive cocci in the microscopic examination (p value <0.005). In the culture method, increased amounts of *Klebsiella*, *Escherichia coli,* and *Citrobacter* spp. were characterized in the patients with gastroenteritis compared with the control group. There was no significant difference between each group of patient and healthy controls regarding the prevalence of the *Pseudomonas aeruginosa*, *Clostridium difficile, Proteus *spp*. *and* S. aureus* (p value >0.005)*. *Indeed, there was no significant difference between each group of patient and healthy group regarding the prevalence of the mucus secretion, epithelial cell, and yeast in the smear (p value > 0.005). 

**Table 2:** Diversity of fecal bacteria in patients with IBS and gastroenteritis and control group

Bacterial species, (n)^a^	IBS (80)	Gastroenteritis (80)	Control (50)	*p* value
Culture results				
*Escherichia coli*	73	56	48	0.001
*Klebsiella *spp*.*	9	21	0	0.001
*Enterobacter *spp*. *	7	8	0	0.73
*Citrobacter *spp*.*	10	15	0	0.006
*Pseudomonas aeruginosa*	2	1	0	0.498
*Clostridium difficile* *Proteus *spp. *S. aureus*	2 1 2	0 1 5	0 0 1	0.194 0.498 0.346
Microscopic Findings^b^				
Enterotype 1 (*Bacteroides* spp*.*)	2	2	2	0.857
*Lactobacillus* spp.	29	13	7	0.002
*Actinomycetes *	12	2	2	0.006
Gram positive cocci (aerobic and anaerobic)	14	41	19	0.01
*Bifidobacter* spp.	0	1	0	0.442
Spore forming bacteria	31	30	12	0.139
Gram negative bacilli (aerobic and anaerobic)	42	47	43	0.001
*Fusobacterium* spp.	4	2	0	0.243
White blood cells Red blood cells Mucus secretion Epithelial cell Yeast	6 0 20 37 23	22 8 30 41 34	0 1 15 18 19	0.001 0.005 0.229 0.313 0.186

Enterotype 1 was indicated when *Bacteroides* species constitute major population of faecal microbiota compared with other bacteria commonly present in the human intestine. Reports of the specified microbes and cells were done based on their distinct morphologies in the microscopic examination.

a N, numbers of total patients and control samples is shown in each category. Colonization and infection of the samples with defined bacterial species and genera are depicted in each column as numbers of the positive samples.

## Discussion

Irritable bowel syndrome was characterized by abdominal pain, bloating and changes in habit. Although, the pathophisiology of IBS is still unclear, but Visceral hypersensitivity, disturbed intestinal reflexes, psychological disorders, gastrointestinal infection and an imbalance of gut microbiota are the main proposed risk factors that are involved in the pathogenesis of IBS (8). Microbial dysbiosis of the gut is thought to be involved in the pathogenesis of IBS through the facilitating adhesion of pathogens to the bowel wall (9). In general, gut microbiota is classified into different enterotypes. An enterotype is a classification of living organisms based on its bacteriological ecosystem in the gut microbiome. All humans can be divided into one of three discrete gut enterotypes based on the composition of the microbiota that have no relation to nationality, age, sex, and other characteristics (10). In our study, we found an increase in the frequency of Enterobacteriaceae members such as* E.coli *and* Citrobacter *spp*. *by the culture method and microscopic examinations among the IBS patients. This finding was similar to the report of Si, *et al*. and Carroll, *et al*. that showed a significant increase for Enterobacteriacea among the IBS patients compared with the control group  (11;12). Almost all of our IBS patients had bloating (92.5%). An overgrowth of some of gram negative bacteria such as *Citrobacter* and *Klebsiella *has been identified as a cause of bloating (13-15). In the Faber, *et al.* study, an overgrowth of *citrobacter freundi* and *klebsiella pneumonia *in patients with IBS was reported in 19% and 22%, respectively (15). His result showed an improvement in symptoms of abdominal pain (62%) and bloating (73%) after supplementation with antibiotics and exogenous flora. According to our results*, *it seems that there is an association between overgrowth of *Citrobacter* spp. and bloating. In a study by Mättö, *et al*., no difference in the prevalence of spore-forming bacteria, Lactobacilli, Enterococci and yeasts was observed between the IBS and control groups, whereas slightly higher numbers of coliforms, as well as an increased aerobe: anaerobe ratio was observed in the IBS group  (16). The high prevalence of *Lactobacillus* spp. in our patients' fecal samples was also previously reported by Tana, *et al. *(9). However, contradictory results that showed no difference in the prevalence of Lactobacilli between the IBS and the control groups were documented. Other work by Jeffery and colleagues found subgroups among the patients with IBS with varying microbial signatures. However, generally an increase in the Firmicutes to Bacteroidetes ratio was evident in patients with IBS who differed from normal populations (17). Results of our study and those that were obtained by other researchers suggested a link between the gut microbiota and IBS, which could lead to the design of therapeutic options (18). Since many of the GI tract microbes have not yet been cultured and are only recognized based on their 16S rDNA sequences, molecular high-throughput approaches have been developed to study the diversity and functionality of the thousands of phylotypes that have been predicted to be present in the GI tract (19). 

Results of this study proposed an association between the presence of *Citrobacter* spp., *Klebsiella* spp. and Actinomycetes and the occurrence of IBS or gastroenteritis. Depletion of gram-positive cocci and gram-negative bacilli in these patients compared with the control samples also suggested dysbiosis of intestinal microbiota in these patients.
